# Diverse imaging features of adolescent glioblastoma

**DOI:** 10.1259/bjrcr.20210207

**Published:** 2021-12-17

**Authors:** Thomas Campion, Sara Stoneham, Ayisha Al-Busaidi, Atul Kumar, Zane Jaunmuktane, Sebastian Brandner, Neil Kitchen, Stefanie Thust

**Affiliations:** 1 Lysholm Department of Neuroradiology, National Hospital for Neurology and Neurosurgery, London, UK; 2 Teenage and Young Adult Cancer Unit, Department of Paediatric Oncology, University College London Foundation Hospital, London, UK; 3 Division of Neuropathology, National Hospital for Neurology and Neurosurgery, University College London NHS Foundation Trust, London, UK; 4 Department of Clinical and Movement Neurosciences and Queen Square Brain Bank for Neurological Disorders, Queen Square Institute of Neurology, University College London, London, UK; 5 Department of Neurodegenerative Disease, Queen Square Institute of Neurology, University College London, London, UK; 6 Department of Neurosurgery, National Hospital for Neurology and Neurosurgery, London, UK; 7 Neuroradiology Academic Unit, Department of Brain, Repair and Rehabilitation, UCL Institute of Neurology, London, UK; 8 Imaging Department, University College London Foundation Hospital, London, UK

## Abstract

We highlight an unusual case of multifocal glioblastoma in an adolescent patient, manifesting as four discrete brain lesions, each distinct in appearance. Familiarity with the diverse imaging features of glioblastoma can reduce misdiagnosis and avoid treatment delays.

## Clinical presentation

**Figure 1.**
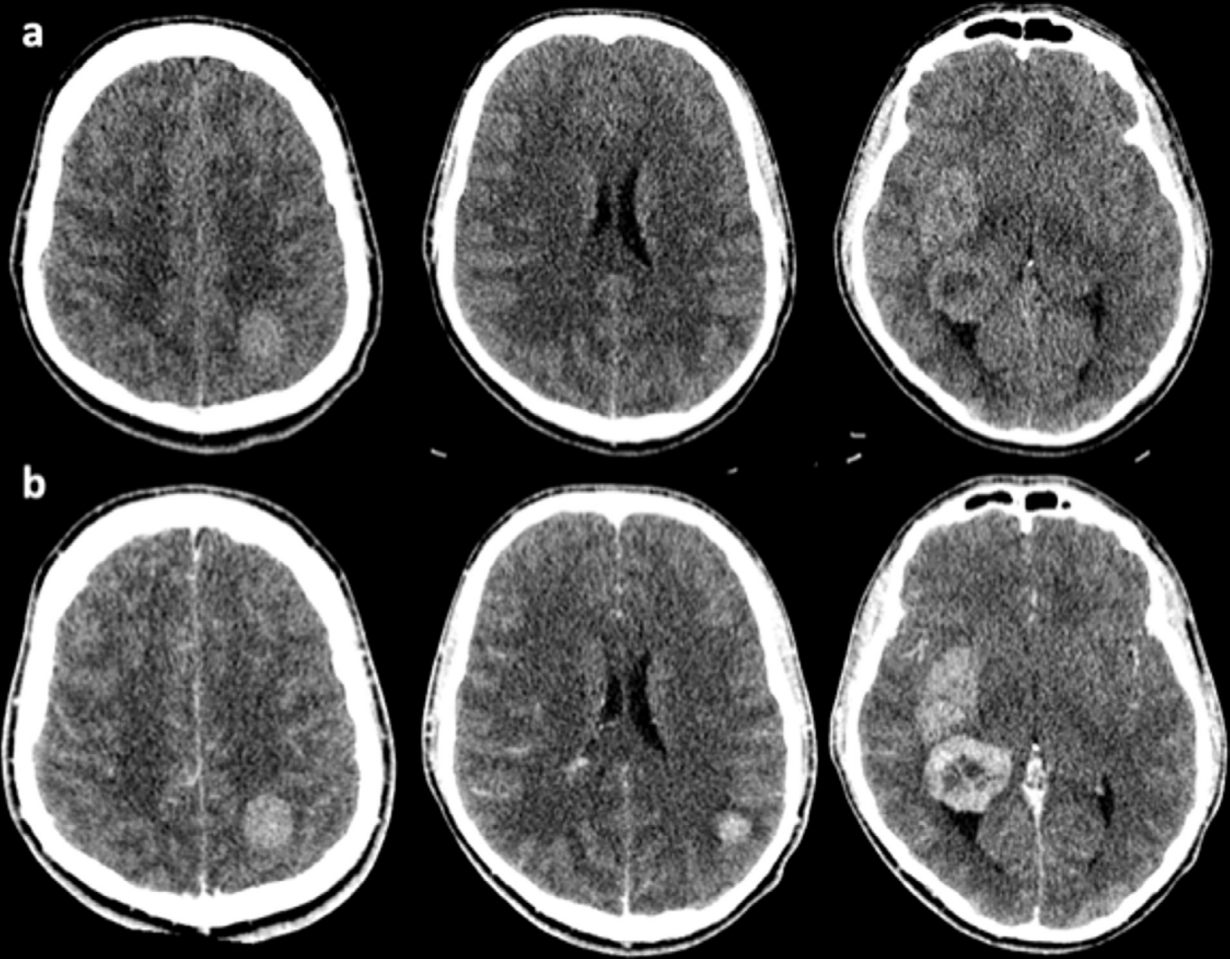
Pre- (**a**) and post-contrast (**b**) CT imaging performed on admission demonstrating bilateral cerebral tumours.

A 17-year-old, previously well male was admitted to the emergency department with a 2-week history of progressive headaches, nausea and vomiting. This was followed by a single seizure with transient loss of consciousness. CT brain imaging performed on admission demonstrated several masses in both cerebral hemispheres (Figure 1). CT imaging of the chest, abdomen and pelvis identified no abnormality, and serum tumour markers (beta-HCG and alpha-fetoprotein) were negative.

## MR imaging findings

**Figure 2.**
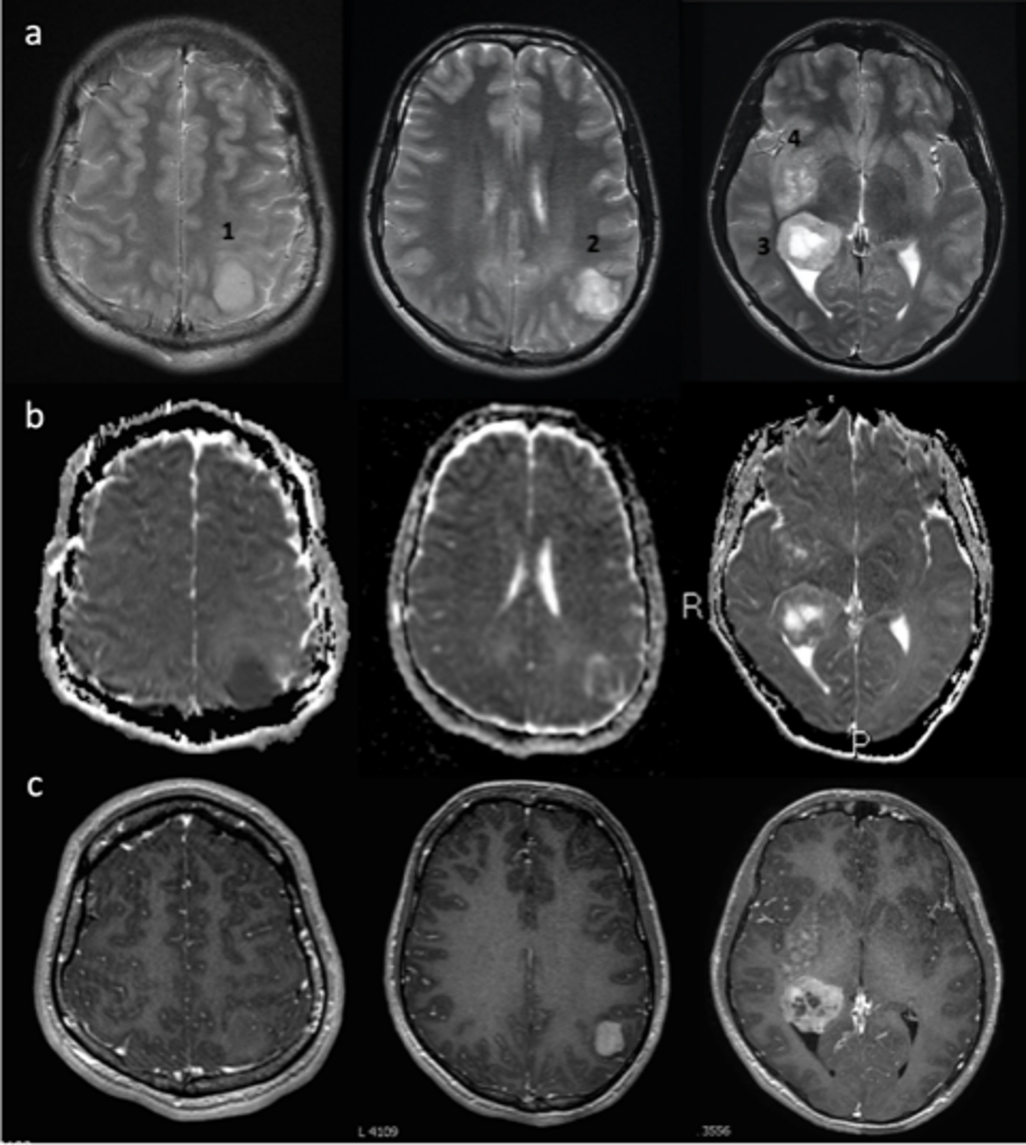
T2 (**a**), ADC (**b**), T1+Gad (**c**) images showing multiple masses: Lesion 1 is circumscript with marked diffusion restriction and no significant Gadolinium enhancement. Lesion 2 is heterogenous featuring a rim of oedema/infiltration, ADC values similar to surrounding brain and avid enhancement. Lesion 3 exhibits rim enhancement and central necrosis. Lesion 4 is poorly marginated on all sequences demonstrating mild patchy Gadolinium uptake. ADC, apparent diffusion coefficient.

Four tumours varying in size and morphology were present within both cerebral hemispheres (Figure 2): Lesion 1 in the left superior parietal lobule appeared circumscript and markedly T2 hyperintense, showing restricted diffusion without Gadolinium contrast enhancement. Lesion 2 in the left inferior parietal lobule exhibited diffusivity similar to surrounding brain parenchyma and solid contrast enhancement. Lesion 3 was the largest, located at the right dorsal thalamic border. Because this lesion was centred on the lateral ventricular ependymal margin, it appeared partially intraventricular. Lesion 3 demonstrated avid peripheral contrast uptake with central necrosis. Lesion 4 consisted of ill-defined T2 hyperintense expansion of the right lentiform nucleus and surrounding brain parenchyma with patchy contrast uptake. Localised perilesional T2 signal abnormality (oedema ± infiltration) ranged from minimal to diffuse, with areas of normal appearing brain interposed between the lesions.

## Therapeutic management

**Figure 3.**
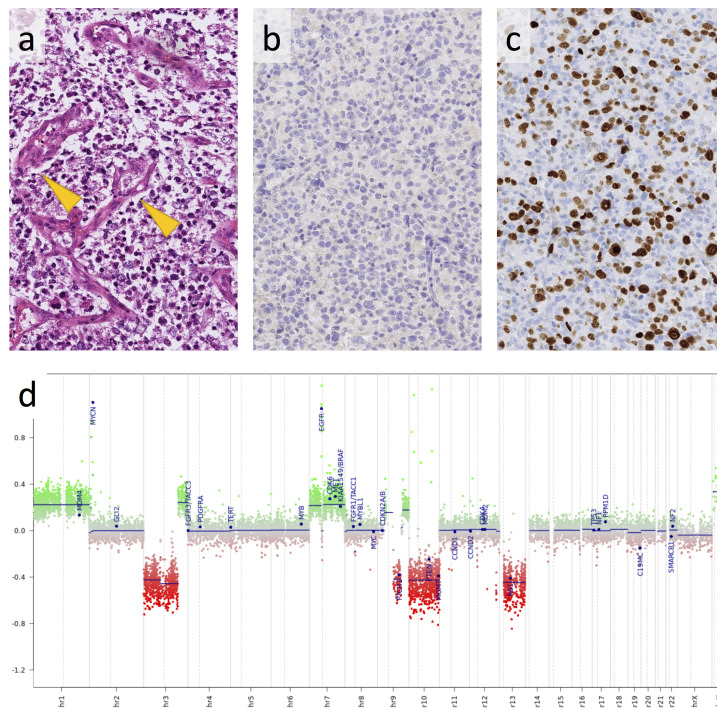
Histology of the tumour shows a monomorphic population of neoplastic glial glial cells with frequent microvascular proliferations (arrow) (a). Immunoreactivity for mutant IDH1 (R132H) is negative (b) and ki67 immunostain for proliferating tumour cells shows a very high labelling index (c). The copy number profile, derived from the Illumina 450 K methylation array, shows the characteristic chromosome seven gain, 10q loss, and amplification of MYC and EGFR (d). The scale bar corresponds to 200 µm in (a)–(c). EGFR, epidermal growth factor receptor.

The patient was transferred to the teenage and young adult (TYA) cancer unit, and a biopsy was performed. Histological examination showed morphological evidence of glioblastoma (WHO Grade IV). Post-operatively, the patient suffered a rapid clinical deterioration with increasing headaches, vomiting, blurred vision and new left arm paraesthesia and was commenced on steroids to help alleviate these symptoms. Upon histopathological assessment, immunohistochemical tests showed absence of the most common mutation of isocitrate dehydrogenase (IDH) 1 (R132H) (Figure 3), retained *Alpha*-thalassaemia X-linked mutant retardation (ATRX) protein expression in tumour cell nuclei, and a very high Ki67 proliferation index (Figure 3). On molecular pathological analysis,^1^ the tumour was negative for rare IDH1 and for IDH2 (R172) mutations, BRAF (V600), or Histone 3.3 (K27, G34) mutations. Epidermal growth factor receptor was amplified (18 copies) and the methyl guanine methyl transferase (MGMT) promoter was unmethylated. The unusual presentation of a tumour, which is most commonly found in adults in their fifth-seventh decade, prompted us to perform a methylation array with subsequent algorithmic classification.^1^ This investigation confirmed the presence of a classical IDH wild-type glioblastoma with the methylation subclass receptor tyrosine (RTK) kinase II.^2^^3^ The copy number profile (Figure 3) showed in addition a MYC amplification.

As a patient with this histological diagnosis and extent of disease is unlikely to be cured by resection, *i.e*. when the lesions involve multiple sites and are embedded in deep structures, rapid arc intensity-modulated targeted radiotherapy (54 Gy) was administered alongside adjuvant temozolomide, with palliative intent.

Initially, a partial radiological response to treatment was observed, with complete symptom resolution. However, at 3 months into treatment a new gadolinium enhancing lesion developed in the dorsal medulla, indicating progressive disease. This new deposit and one of the previously treated cerebral lesions continued to grow. Temporary effective symptom control was achieved with further focal radiotherapy to the site of new disease, but disseminated ependymal and parenchymal disease followed, with death at 11 months post diagnosis.

## Discussion

Glioblastoma is a relatively uncommon diagnosis in adolescence,^4^ and it may be considered low on the differential list - more so if the imaging findings are perceived as atypical. We would like to draw attention to this case, in which the coexistence of several tumour morphologies could create a diagnostic dilemma. A learning point is to consider the possibility of malignant glioma in cases of multiple brain masses with different imaging appearances.^5^

The diverse post Gadolinium (non-, solid-, rim-enhancing), *T*_2_ weighted (circumscript *vs* diffuse) and diffusion-weighted (restricted, intermediate, facilitated) features encountered in this case are all known to exist within the phenotypic spectrum of glioblastoma,^6,7^ but may be confused with other entities, even dual pathology, when occurring in combination.

Multifocal cerebral lesions may raise concern for infective-inflammatory aetiologies, especially in a young individual, metastatic disease or lymphoma.^8^ Importantly, rapid progression can occur in glioblastoma and should not deter radiologists from suspecting the diagnosis.^9^ Physiological MR imaging (perfusion, spectroscopy) can aid the diagnosis of glioblastoma,^10^ however, in this case tissue diagnosis was prioritised because of the fulminant clinical decline.

In adult patients, multiple intracranial tumours are present at diagnosis in up to 34% of glioblastomas.^11^ It is probable that diverse glioblastoma morphologies represent different stages of the disease. Moreover, evidence exists for significant genetic heterogeneity amongst synchronous tumours^12^ as well as within the same glioma through mutational evolution over time^13^ ; glioblastomas have been shown to originate up to seven years before diagnosis and acquire most of their driver mutations leading to genetic heterogeneity in this period (*i.e.* before treatment is initiated).^14^

Tumours involving the subventricular zone, as the presumed site of neural stem cell origin of glioblastoma, have been postulated to be more often multifocal and to recur at distant sites^15^ ; in our case. the two lesions most closely related to the ventricular surface represented the sites of recurrence and progression.

Multifocal glioblastoma typically have the IDH-wildtype genetic signature^16^ associated with poor prognosis and limited response to maximum therapy. Multifocal disease may further shorten the dismal survival of glioblastoma, if gross total resection is difficult to achieve.^17,18^ At present, most high-grade gliomas in adolescence undergo standard glioblastoma therapy, consisting of radiation and chemotherapy (Temozolomide). In the future, rapid genetic and epigenetic tumour profiling may improve focused approaches,^19^ for which strategies in development include immune modulation and therapies directed against specific signalling pathways.^20^

## Conclusion

Glioblastoma should be considered as part of the differential diagnosis in young patients presenting with multiple brain masses. The disease may exhibit variable MR imaging features, which can coexist in the same individual.

## Learning points

- Glioblastoma should be considered in young patients presenting with multiple brain masses.
- Glioblastoma manifests with variable MR imaging features, which can coexist in the same individual.
- Imaging findings include enhancing and non-enhancing lesions, with or without diffusion restriction.
